# Clinical outcomes following mobile-bearing unicompartmental knee arthroplasty in patients with varying degrees of perioperative alignment change

**DOI:** 10.1186/s42836-025-00363-6

**Published:** 2026-01-20

**Authors:** Genbin Wu, Xinmeng Jin, Jinwei Chen, Zhongwei Sun, Peng Miao, Haifeng Zhang, Yinxian Yu

**Affiliations:** 1 Department of Orthopedic Surgery, Shanghai General Hospital, Shanghai Jiao Tong University School of Medicine, Shanghai, 200240 China; 2 Department of Orthopedic Surgery, Tongzhou District People’s Hospital of Nantong, Jiangsu Province, Nantong, 226300 China

**Keywords:** Unicompartmental knee arthroplasty, Perioperative varus deformity change, Mechanical alignment

## Abstract

**Background:**

Although favorable survival and good outcomes have been reported with Oxford unicompartmental knee arthroplasty (UKA), the effect of perioperative alignment change on patient-reported outcome measures (PROMs) remains controversial. In this study, we investigated the impact on outcomes and survivorship of medial UKA with significant perioperative alignment changes.

**Methods:**

We retrospectively reviewed 316 patients with anteromedial OA who underwent primary Oxford UKA. The patients were divided into three groups (A, *n* = 146; B, *n* = 98; C, *n* = 72), Group A: mild varus alignment change (≤ 4°), Group B: moderate varus alignment change (> 4° and < 7°) and Group C: large varus alignment change (≥ 7°). The mean follow-up period was 2.9 years (range: 1.9–4.5 years). Patient history, as well as pre- and post-operative KOOS-JR (Knee Injury and Osteoarthritis Outcome Score for Joint Replacement) scores and Kujala scores, were obtained through chart review. Continuous data were compared using analysis of variance (ANOVA). Chi-squared tests were used to compare discrete variables. Linear regression was conducted to estimate the effect of alignment change on the improvement of the KOOS-JR score.

**Results:**

In all groups, the KOOS-JR and Kujala scores showed significant improvement after surgery. At the 1-month follow-up, the difference in mean KOOS-JR score between the groups was not significant (*P* > 0.05). The Kujala score of Group A was highest (*P* < 0.05), and the difference between Group B and C was not significant (*P* > 0.05). In the 2-year follow-up, mean KOOS-JR and Kujala outcomes were comparable among groups (*P* > 0.05). The KOOS-JR MCID in each group was 71% in Group A, 73% in Group B, and 85% in Group C. Linear regressions showed no statistically significant relationship between the variation of perioperative alignment and KOOS-JR scores (*P* > 0.05). The 2-year survival rate for the entire cohort was 100%.

**Conclusion:**

UKA with a low angle of perioperative varus deformity change would have a rapid improvement of functional scores, especially the relief of anterior knee pain. Severe varus deformity with large perioperative alignment change can still obtain desirable outcomes.

Video Abstract

**Supplementary Information:**

The online version contains supplementary material available at 10.1186/s42836-025-00363-6.

## Introduction

Knee osteoarthritis (OA) is the most common arthropathy, affecting the elderly population [1, 2]. The progress of degeneration does not affect all knee compartments equally. The medial compartment is 10 times more at risk of suffering from OA compared with the lateral compartment [2–4]. Unicompartmental knee arthroplasty (UKA) is widely accepted as an effective and durable alternative to isolated unicompartmental knee arthritis [5–7].

Favorable clinical outcomes, high survivorship, and rapid postoperative recovery after Oxford UKA arthroplasty have been consistently reported [8–10]. UKA is characterized by minimal invasiveness, reduced blood loss, and preservation of most native knee structures, resulting in effective pain relief. Since medial UKA preserves the anterior cruciate ligament (ACL) and accepts only insignificant lateral compartment OA and asymptomatic patellofemoral degenerative changes, this procedure better preserves native joint kinematics and proprioception, but some pre-existing degeneration of the lateral and patellofemoral compartments may be acceptable [9, 11].

Although mobile-bearing UKA is an established procedure, the association between perioperative alignment changes and outcomes remains controversial. Unlike fixed-bearing UKA, the design of the Oxford mobile-bearing UKA emphasizes restoring natural knee biomechanics rather than pursuing a “perfect neutral” mechanical alignment and which enables it to tolerate greater deformity changes [12, 13].

Traditionally, mechanical alignment has served as a valuable approach that aimed to evaluate the mechanical axis following medial UKA. In many studies [9, 14], postoperative alignment with mild varus has been recommended. Overcorrecting might accelerate osteoarthritis progression in the contralateral compartment [15]. The conventional concept of medial UKA arthroplasty has been challenged in other studies. Recent studies have proposed some perspectives that patients who have significant flexion contractures or varus alignment > 15 degrees can be tolerated with UKA and obtain excellent functional outcomes [16, 17]. OA patients in China exhibit significant variability in disease severity, particularly in coronal alignment phenotypes [18, 19]. With the progress of surgical technique and modern instruments such as the surgical navigation system and robotic-assisted technique, UKA indications were allowed to expand [20, 21].

Currently, most knees would obtain slight varus alignment following the conventional strategies of Oxford UKA. However, patients with severe varus deformity might experience great varus alignment. According to the traditional concept of Oxford UKA, overcorrecting may compromise both knee kinematics and function after medial UKA. The present study aimed to determine if great varus alignment change following Oxford UKA arthroplasty is acceptable or not, and whether greater or less coronal alignment change would yield better clinical outcome and implant survivorship at mid-term follow-up.

## Material and methods

### Patients and study design

**Fig. 1:**
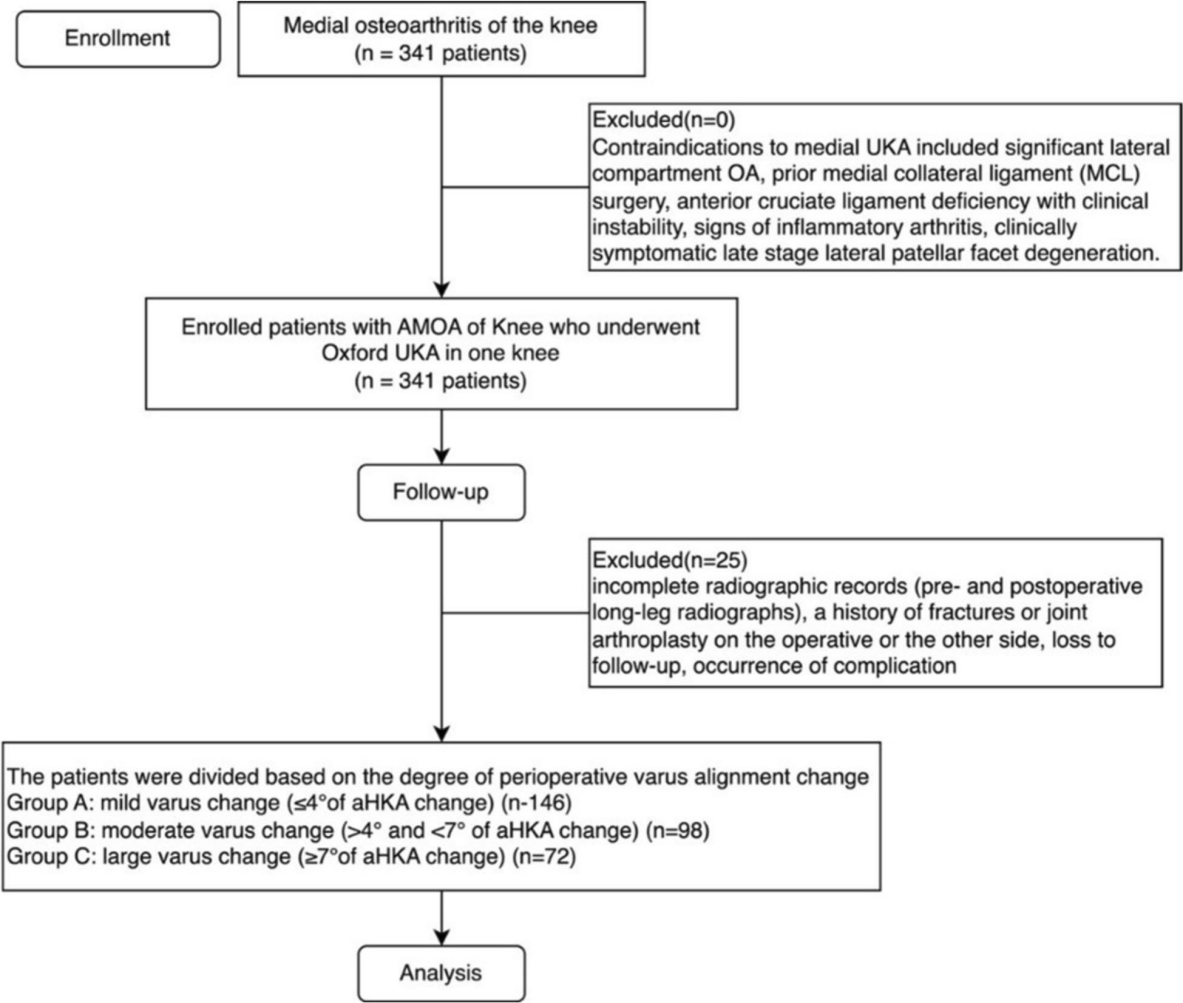
Flowchart of patient inclusion and exclusion

After institutional review board approval, a retrospective cohort study was conducted on 316 consecutive patients (Table 1) who underwent medial mobile-bearing UKA (Oxford® UKA; Zimmer Biomet, Inc., Warsaw, IN, USA) in one knee from Feb 8th, 2018, to December 20th, 2023, by a surgery specialist. A flowchart describing patient inclusion and exclusion is presented in Fig. 1.

**Table 1:** Demographics of the patients

Characteristic	Total (316)			*P*-value
Mean age in years (range)	69.0 ± 12.9 (52–85)			
Sex, *n* (% of group)				
Men	96			
Women	220			
Mean Body mass index (range)	Group A	Group B	Group C	
	24.6 ± 2.7	25.3 ± 2.6	25.4 ± 2.8	> 0.05
Kellgren-Lawrence grade (medial compartment)				> 0.05
K-L grade 2	8 (5%)	2 (2%)	0	
K-L grade 3	34 (23%)	20 (20%)	12 (16%)	
K-L grade 4	107 (72%)	76(78%)	60 (84%)	
Pre-Op KOOS-JR	64.5 ± 7.9	61.8 ± 9.5	58.6 ± 5.2	< 0.0001
Pre-Op Kujala	64.3 ± 7.2	61.3 ± 8.0	58.4 ± 5.7	< 0.0001
Operative time (mins)	72.0 ± 9.1	70.6 ± 7.6	75.2 ± 10.0	> 0.05
Blood loss (mL)	118.4 ± 19.1	124.8 ± 16.3	120.5 ± 18.8	> 0.05
Length of stay (days)	3.7 ± 0.4	3.5 ± 0.5	3.5 ± 0.4	> 0.05

The inclusion criteria for medial UKA at our institution were based on clinical and radiographic findings of symptomatic end-stage isolated anteromedial osteoarthritis. Patients were eligible for medial Oxford UKA if they had passively correctable varus deformity to < 15° and fixed flexion deformity of < 10°. Contraindications to medial UKA included significant lateral compartment OA, prior medial collateral ligament (MCL) surgery, anterior cruciate ligament deficiency with clinical instability, signs of inflammatory arthritis, and clinically symptomatic late-stage lateral patellar facet degeneration.

### Data collection and outcome measures

Data were collected on a standard case record form. The recorded baseline patient characteristics included age and sex and the knee side of UKA, body mass index (BMI), degree of varus deformity, and ROM. All patient data were de-identified prior to analysis to ensure confidentiality. The data were stored on a password-protected, encrypted institutional server with access restricted to the principal investigators and authorized staff only.

Patients were followed at 1 month, 3 months, 6 months, and 1 year and then annually thereafter. The data for this study were primarily extracted from the preoperative, 1-month, and 2-year postoperative time points. A defined data collection window of ± 2 weeks was allowed for the 1-month visit, and ± 1 month for the 2-year visit to accommodate clinical scheduling variability. Patient-reported outcome measures (PROMs), including the Knee Injury and Osteoarthritis Outcome Score for Joint Replacement (KOOS-JR) [22] and Kujala scores [23], were collected prospectively as part of routine clinical care. Complications, such as infection, component loosening, fractures, and bearing dislocations, were also recorded.

The study termination date for inclusion was December 20, 2023. This date was chosen to ensure all included patients had the opportunity to reach at least the 2-year follow-up milestone, which served as the primary endpoint for the mid-term outcome analysis. And the primary endpoint for this study was the KOOS-JR score at the 2-year postoperative follow-up. Secondary endpoints included the Kujala score at all timepoints. Patients were excluded if they had incomplete radiographic records (pre- and postoperative long-leg radiographs), a history of fractures or joint arthroplasty on the operative or the other side, loss to follow-up, occurrence of complications.

### Radiographic assessment protocol

Radiographic assessment was performed using anteroposterior (AP), lateral standing, and standardized long-leg standing radiographs at each time point. The radiographic measurements (Anatomical Hip-Knee-Ankle Angle, aHKA) were conducted independently by three senior radiologists who were blinded to the clinical outcomes of the patients to minimize assessment bias. The preoperative measurements were conducted after the patient was listed for surgery but prior to the operative date. The postoperative measurements were performed on radiographs taken during the standard 6-week follow-up visit. All measurements for a given patient (pre- and post-operative) were conducted by the same assessor in a single session to ensure consistency, but the assessors were blinded to other results. This standardized radiographic protocol was employed for all patients.

To quantify the reliability of the measurements, we calculated both inter- and intra-rater reliability. For inter-rater reliability, a random sample of 50 pre-operative radiographs was measured by all three radiologists. The results showed excellent agreement, with an intraclass correlation coefficient (ICC) of 0.92 (95% CI: 0.87 to 0.95). For intra-rater reliability, the same sample of 50 radiographs was re-measured by the primary radiologist after a 4-week interval to minimize recall bias. The agreement was also excellent, with an ICC of 0.95 (95% CI: 0.91 to 0.97).

**Fig. 2:**
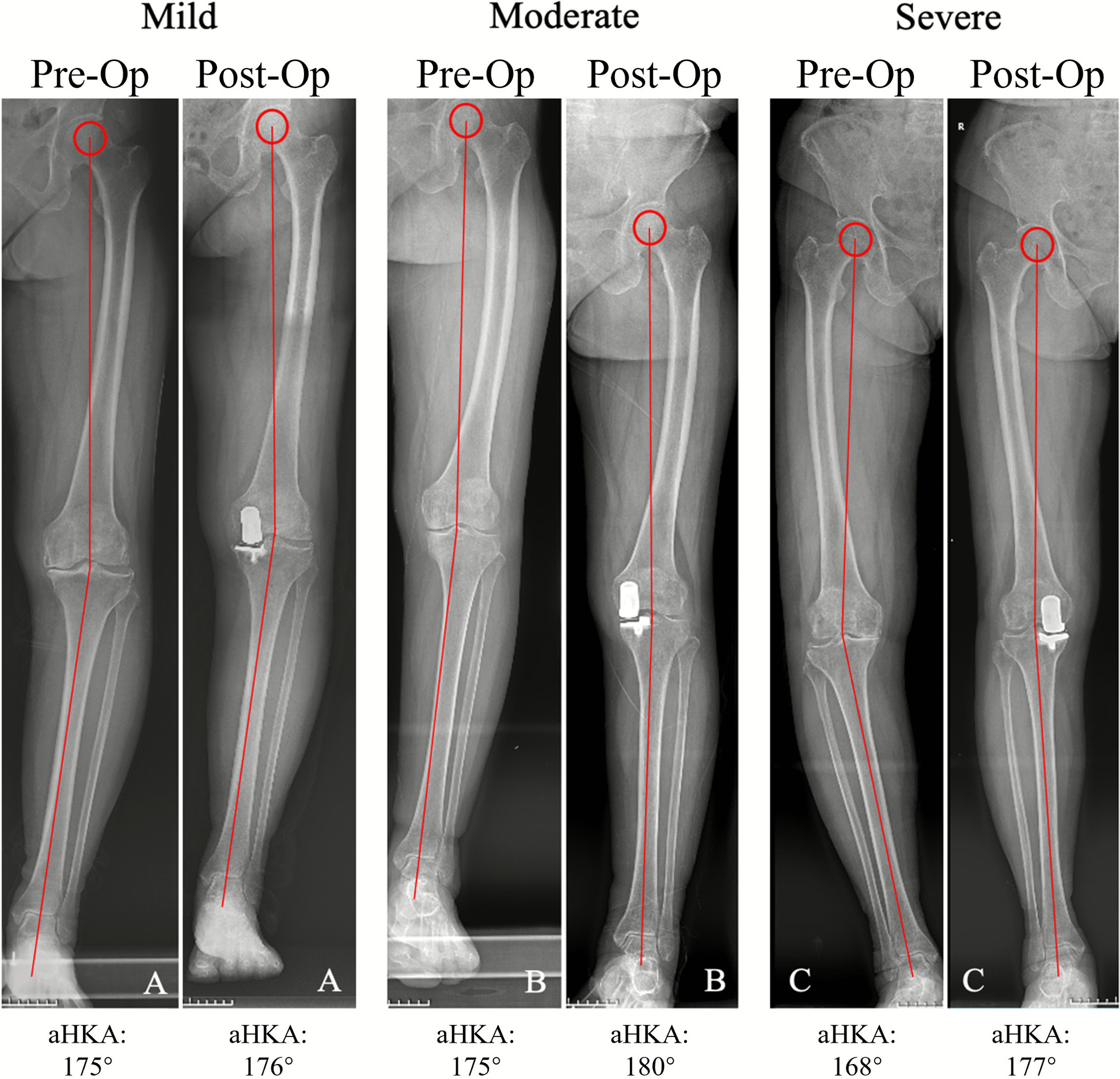
Examples of radiographic assessment of aHKA from different peri-operative varus alignment changes: A: mild varus alignment change (≤ 4°), B: moderate varus alignment change (> 4° and < 7°), C: large varus alignment change (≥ 7°)

The patients were divided into three groups based on the degree of perioperative varus alignment change. The anatomical hip-knee-ankle angle (aHKA) was used to quantify the alignment change. The aHKA was measured on full-length standing radiographs preoperatively and postoperatively, and the change in aHKA (postoperative aHKA minus preoperative aHKA) defined the change angle for each patient. The groups were stratified as follows: Group A: mild varus alignment change (≤ 4°of aHKA change), Group B: moderate varus alignment change (> 4° and < 7° of aHKA change), and Group C: large varus alignment change (≥ 7°of aHKA change). The cutoff of ≥ 7 for defining “large” alignment change was based on Kleeblad et al.’s threshold for substantial varus deformity, while the intermediate categories were established considering that most Oxford UKA cases demonstrate alignment changes of 3°–5° in the literature. The examples of radiographic assessment of different perioperative varus alignment angles are presented in Fig. 2.

### Data analyses

Categorical variables were presented as counts and percentages. Continuous variables were presented as means and standard deviations. A *P*-value less than 0.05 was considered significant throughout the investigation.

The normality of the distribution for all continuous outcome variables, including KOOS-JR and Kujala scores, was formally assessed prior to statistical testing. This assessment was conducted using the Shapiro–Wilk test and supported by visual inspection of Quantile–Quantile (Q-Q) plots. For the dataset used in this study, all variables of interest did not show significantly deviated from normality (Shapiro–Wilk test, *P* > 0.05; Q-Q plots exhibited an approximately linear pattern). Therefore, parametric tests were deemed appropriate and were employed for all subsequent analyses. These groups were assessed by analyses of variance (ANOVA) for differences in KOOS-JR values and Kujala values. Post-hoc pairwise comparisons following ANOVA were conducted using Tukey’s HSD test. The Minimal Clinically Important Difference (MCID) for the Knee Injury and Osteoarthritis Outcome Score for Joint Replacement (KOOS-JR) was used to determine the proportion of patients in each group who achieved a clinically meaningful improvement from pre-operation to the 2-year postoperative visit. The MCID for the KOOS-JR was defined as a change of 16 points. This threshold was not derived from our dataset but was adopted from the validated, anchor-based value established by Dekhne et al. [24]. In their large cohort study, this value was determined using a receiver operating characteristic (ROC) curve analysis, anchored on a single-item Patient Acceptable Symptom State (PASS) question, providing a robust and patient-centered benchmark for meaningful change. A patient was classified as having “achieved the MCID” if their KOOS-JR score increased by 16 points or more between the preoperative and the 2-year postoperative assessment. The proportion of patients achieving the MCID was calculated for each of the three alignment change groups (Groups A, B, and C). Differences in the rates of MCID achievement and K-L grades among the three groups were compared using a Chi-squared test. A *P*-value of less than 0.05 was considered statistically significant.

Statistical analyses were performed with SPSS version 18 software (IBM, Armonk, NY, USA).

### Surgical technique

The patients were anesthetized via general anesthesia. In all patients, a thigh tourniquet was inflated to 240 mmHg. An anteromedial skin incision was made from the upper pole of the patella to the medial aspect of the tibial tubercle. A mini midvastus approach was applied in all cases for the prevention of patellar maltracking [25]. The patella was slightly subluxated laterally but was not everted.

The tibia was first cut by applying the tibial saw guide parallel to the long axis of the tibia at the medial edge of the ACL insertion and to create a tibial slope of 7°.

A horizontal resection was made to remove the worn medial cartilage and a minimal amount (typically 2 mm) of underlying bone. The horizontal resection was perpendicular to the mechanical axis. The posterior condyle of the femur was then cut using intramedullary (IM) femoral-guided instrumentation that connected the femoral drill guide with the intramedullary (IM) link. The flexion gap was set at 100° of flexion, and the extension gap was set at 20° of flexion. The distal condyle of the femur was cut using the milling technique to create an equal flexion–extension gap. All operations used the same instrumentation. A cocktail mixture (5 mg Morphine, 0.5% Ropivacaine hydrochloride 150 mg, methylprednisolone 10 mg, Normal Saline (0.9%) 50 mL) was systematically infiltrated into the periarticular tissues. No drain and a Foley catheter were used in the surgery. The operative time, blood loss, and intraoperative fracture were recorded.

### Postoperative protocol

The surgical wound was cleaned. Intravenous fluids were stopped. Patients were allowed to begin partial weight-bearing and active-assisted ROM knee exercises. If an aseptic inflammatory reaction, like swelling, redness, warmth around the incision, a cold pack would be applied. The patients were discharged from the hospital on postoperative day 2 if they did not need intravenous medication to control acute postoperative pain. They were taught to practice ambulation for 10 min two times daily and perform quadriceps exercises, with approximately 20 repetitions three times daily, and were instructed to begin ankle pump exercises as soon as possible to reduce the risks of deep vein thrombosis (DVT) and pulmonary embolism (PE). At each follow-up, patients were assessed with knee motion, muscle strength, signs of DVT, such as swelling of the leg, pain in the leg, redness, and increased warmth; no DVT screening test was performed routinely. Patients with clinically suspected DVT underwent leg ultrasound and, if confirmed, were treated appropriately by the hematologist.

## Results

At the beginning, 341 patients were enrolled in this study, but 25 of them were excluded because they underwent contralateral knee arthroplasty during the follow-up period. A total of 316 patients who underwent the Oxford UKA arthroplasty were evaluated in this study at last. The 316 (220 females) identified patients had a mean age of 69.0 ± 12.9 years and a mean BMI of 25.1 ± 2.7. The BMI scores were comparable among different groups. And the K-L grade of Group C was higher than Group A (*P* < 0.05); the differences between Group A and Group B, Group B and Group C were not significant (*P* > 0.05) (Table 1).

Analysis of KOOS-JR MCID achievement is presented in Table 2. Group A included 146 knees (46.2%), Group B included 98 knees (31.0%), and Group C included 72 knees (22.8%). There were 80 (25.3%) patients who did not achieve KOOS-JR MCID by the last postoperative visit. Comparison of different angles of alignment change in those who did and did not achieve KOOS-JR MCID is presented in Table 2. Notably, the patients of Group C (large varus alignment change) had numerically higher percentage of KOOS-JR MCID achievement (85%) when compared to Group A (mild varus alignment change, 71%) and Group B (moderate varus alignment change, 73%). The difference was significant (*P* < 0.05).

**Table 2:** Assessment of KOOS-JR MCID achievement by different alignment groups

KOOS-JR MCID achievement	Total num	Achieve MCID num	Rate
Group A (≤ 4°)	146	103	71%
Group B (> 4° and < 7°)	98	72	73%
Group C (≥ 7°)	72	61	85%

### Assessment of perioperative lower limb alignment

The patients were divided into three groups: Group A: mild varus alignment change group (*n* = 146) and Group B: moderate varus alignment change group (*n* = 98), and Group C: large varus alignment change group (*n* = 72) (Table 1). Mean aHKA was 174.3 ± 2.8° preoperatively and 176.3 ± 2.3° postoperatively in Group A, 170.7 ± 1.7° preoperatively and 176.0 ± 1.2° postoperatively in Group B, 167.5 ± 1.3° preoperatively and 175.6 ± 1.1° postoperatively in Group C, respectively. In Group A, the Perioperative aHKA was 2.0 ± 0.9°, in Group B, the Perioperative aHKA was 5.3 ± 0.9°, and in Group C, the Perioperative aHKA was 8.1 ± 1.0°. The Pre-operative aHKA angle of Group C was much varus than Groups A and B (*P* < 0.05). The Pre-operative aHKA angle of Group B was much varus than Group A (*P* < 0.05). The post-operative aHKA angles were comparable among groups (*P* > 0.05) (Table 3).

**Table 3:** Perioperative aHKA angle of different groups

aHKA	Pre-OP	Post-OP
Group A (≤ 4°)	174.3 ± 2.8	176.3 ± 2.3^$^
Group B (> 4° and < 7°)	170.7 ± 1.7^*^	176.0 ± 1.2^$^
Group C (≥ 7°)	167.5 ± 1.3^#&^	175.6 ± 1.1^$^

^*^P < 0.05, Group A vs Group B

^#^*P* < 0.05, Group A vs Group C

^&^*P* < 0.05, Group B vs Group C

^$^*P* < 0.05, vs Pre-OP

### Assessment of perioperative KOOS-JR values

**Fig. 3:**
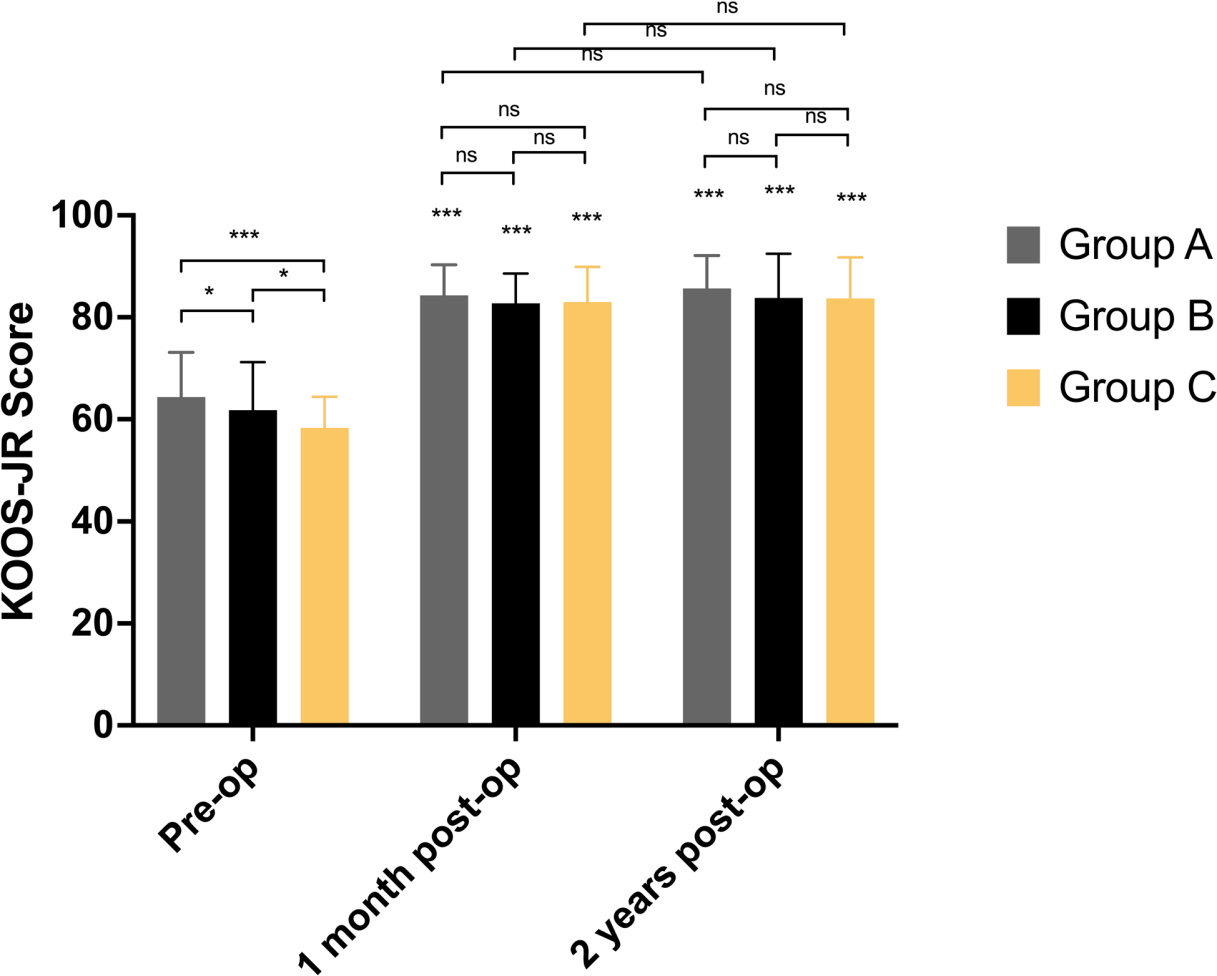
The comparison of the KOOS-JR score from three groups at different time points

The KOOS-JR value of different groups is presented in Table 4. All groups showed significant improvement in 1 month and 2 years postoperatively. Before surgery, Group A got the highest KOOS-JR values (*P* < 0.05). And the KOOS-JR score of Group C was the worst (*P* < 0.05). In the 1-month postoperatively, there was no significant difference in mean KOOS, JR outcomes between groups. In the 2 years postoperatively, mean KOOS, JR outcomes were comparable among groups (*P* > 0.05) (Fig. 3).

**Table 4:** Assessment of KOOS-JR values by different alignment groups

KOOS-JR values	Pre-Op (95%CI)	One month Post-Op (95%CI)	Two years Post-Op (95%CI)
Group A (*n* = 146)	64.5 ± 7.9 (63.2, 65.8)	83.9 ± 5.5 (83.0, 84.8)^$^	85.6 ± 6.0 (84.6, 86.6)^$^
Group B (*n* = 98)	61.8 ± 9.5 (59.9, 63.7)^*^	83.3 ± 5.5 (82.1, 84.4)^$^	84.5 ± 7.3 (83.0, 85.9)^$^
Group C (*n* = 72)	58.6 ± 5.2 (57.4, 59.9)^#&^	83.2 ± 6.6 (81.7, 84.8)^$^	83.6 ± 7.9 (81.8, 85.5)^$^

^*^P < 0.05, Group A vs Group B

^#^*P* < 0.05, Group A vs Group C

^&^*P* < 0.05, Group B vs Group C

^$^*P* < 0.05, vs Pre-OP

### Assessment of perioperative Kujala values

**Fig. 4:**
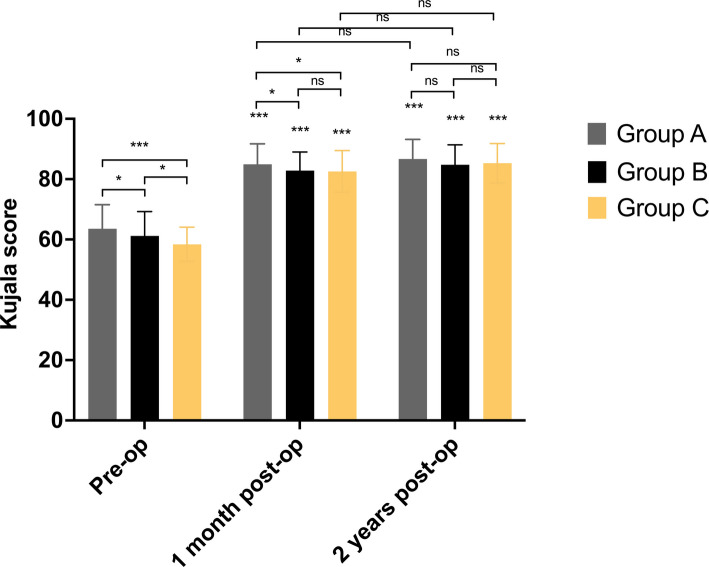
The comparison of the Kujala score from three groups at different time points

The Kujala values of all groups showed significant improvement in 1 month and 2 years postoperatively (Table 5). Before surgery, Group A got the highest Kujala score (*P* < 0.05), and the Kujala score of Group C was the worst (*P* < 0.05). In one month postoperatively, the Kujala score of Group A was still the highest (*P* < 0.05). The difference between Group B and C was not significant (*P* > 0.05). In the 2-year follow-up, the difference in the Kujala score between each group was not significant (*P* > 0.05) (Fig. 4).

**Table 5:** Assessment of Kujala values by different alignment groups

Kujala values	Pre-Op (95%CI)	One month Post-Op (95%CI)	Two years Post-Op (95%CI)
Group A (*n* = 146)	64.3 ± 7.2 (63.2, 65.5)	86.1 ± 5.4 (85.2, 87.0)^$^	86.8 ± 4.6 (86.0, 87.6)^$^
Group B (*n* = 98)	61.3 ± 8.0 (59.7, 62.9)^*^	83.6 ± 5.4 (82.6, 84.7)^*$^	85.6 ± 5.3 (84.5, 86.7)^$^
Group C (*n* = 72)	58.4 ± 5.7 (57.1, 59.7)^#&^	83.0 ± 6.7 (81.4, 84.5)^#$^	84.7 ± 6.2 (83.2, 86.1)^$^

^*^P < 0.05, Group A vs Group B

^#^*P* < 0.05, Group A vs Group C

^&^*P* < 0.05, Group B vs Group C

^$^*P* < 0.05, vs Pre-OP

**Fig. 5:**
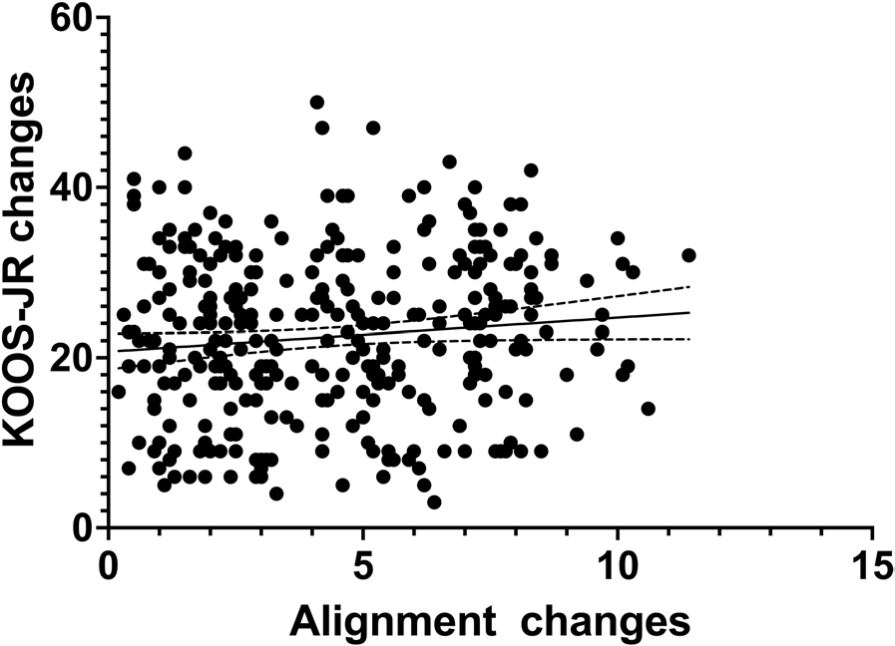
Linear regression analysis between degrees of perioperative varus alignment change and the changes in perioperative KOOS-JR score

The correlation analysis between perioperative alignment changes and the perioperative KOOS-JR score change is presented in Fig. 5. The linear regression analysis revealed that degrees of perioperative varus alignment change were not a significant predictor of the perioperative KOOS-JR score change (Unstandardized Coefficient B = 0.02915, 95% CI: −0.001 to 0.059, *P* = 0.0564).

### Complications

There were no revision cases requiring conversion to TKA. Furthermore, no additional surgery following UKA was required during the study period. There were no cases of bearing dislocation or fatal thromboembolism.

## Discussion

The use of UKA has increased significantly over the past decades, accompanied by growing research interest in clinical outcome parameters and prosthesis survivorship. The results of this study demonstrated that Oxford UKA with a great varus alignment change did not demonstrate inferior Patient-Reported Outcomes or rapid early failure compared with the mild and moderate varus alignment change group in a mean follow-up of 2.9 years.

It is important to note that the Oxford UKA philosophy employs a gap-balancing technique [26], which aims to restore the natural tension of the soft-tissue envelope and native joint kinematics. The perioperative coronal alignment change is therefore a consequence of restoring the medial ligamentous tension and joint line, rather than a preoperatively planned mechanical target. Notably, UKA patients with greater varus angle change demonstrated numerically higher percentage of minimal clinically important difference achievement (MCID). In our study, the group with great varus alignment change (Group C) demonstrated favorable outcomes, which might be attributed to the adaptive characteristics of the Oxford mobile-bearing design.

It is noted that there is no universally accepted threshold for defining a “large” alignment change in Oxford, UKA. Kleeblad et al. [27] in their seminal work on the feasibility of UKA in varus knees, specifically defined “great varus” as > 7° and demonstrated that correction was mechanically achievable in the majority of such cases. According to the literature, the perioperative alignment change following Oxford UKA arthroplasty mainly distributes from 3°–5°. However, during routine clinical practice, we noticed that some AMOA patients might undergo large alignment changes after Oxford UKA, so we artificially determined such angular cutoffs. We define ≥ 7° as a “large” alignment change in our study. In the study of Rahman et al. [20], they similarly found no significant relationship between the degrees of alignment correction and postoperative KOOS-JR scores or MCID achievement.

While existing literature on perioperative alignment correction is based exclusively on a fixed-bearing platform, this study is, to our knowledge, the first to specifically investigate the perioperative alignment change on Oxford mobile-bearing UKA. Unlike mobile-bearing UKA, the design of fixed-bearing UKA might be more sensitive to larger alignment changes due to the lack of a mobile bearing’s congruity [28]. Therefore, our results may not be directly applicable to fixed-bearing UKA.

Interestingly, Group C showed inferior Kujala scores at the 1-month postoperative assessment. There are two primary non-mutually exclusive explanations: the influence of baseline differences and the impact of great varus change on patellofemoral kinematics. A plausible mechanism is that a great varus change could alter the tension of medial soft tissue structures and shift the mechanical axis toward the patient’s natural alignment, which could potentially change patellar tracking and soft tissue adaptation stress. The change in overall limb alignment and subsequent dynamic muscle balance could affect the patellofemoral articulation, especially in the early postoperative period before soft tissues have fully adapted. And patients with severe, long-standing varus have adapted their gait and soft-tissue tensions to that deformity. A great alignment change toward the patient’s natural alignment may place unaccustomed stress shift, contributing to anterior knee pain during early recovery.

The Kujala score is a specific measure of patellofemoral function and anterior knee pain. As previously theorized, the Kujala evaluation system may be more sensitive than the KOOS JR evaluation system for detecting differences in anterior knee pain and function due to its lower ceiling effect [29]. We believe the pre-existing baseline differences between groups are likely the primary confounding factor for this specific finding. Group C had statistically significantly (*P* < 0.05) worse preoperative Kujala scores compared to Group A. It is highly possible that Group C patients underwent surgery with a poorer baseline status of patellofemoral health, which was reflected in their slower recovery as measured by the Kujala score at one month. But these differences were resolved by the 2-year follow-up, suggesting that the influence of surgery is not persistent in the mid-term, which is in accord with the result that there was no significant correlation between perioperative alignment change and MCID achievement.

The middle-aged populations in Asia tend to develop knee osteoarthritis younger age than before [30]. A meta-analysis study indicated that Asia populations frequently engaged in both deep squatting with knee hyperflexion and kneeling during motion. Additionally, domestic studies revealed that a great proportion of middle-aged Chinese individuals, particularly women, exhibit a characteristic body habitus of shorter stature with higher BMI [31]. These factors could explain why Oxford UKA candidates have expanded to younger and more active patients in recent years. Furthermore, these specific young OA patients often present with concomitant severe lower limb varus deformities [32].

UKA candidates presenting with severe preoperative varus deformity may unavoidably obtain greater varus angle change to restore pre-arthritic alignment during surgery. Kleeblad et al. [27] investigated OA patients with substantial varus (> 7 degrees, but < 15 degrees) deformity and found that the majority of patients with severe preoperative deformity achieved a higher mechanical axis correction of 1 to 4 degrees, or 5 to 7 degrees. However, given the distinct anthropometric characteristics and activity patterns of Asian populations, both the angle and frequency of required deformity change may be greater in Asian patients compared to Western patients. Conversely, late-stage OA patients always suffer from long-term severe varus of lower limb alignment. The character of the patient’s gait and the tension of peri-articular soft tissue already adapt to the deformity. The aggressive correction towards neutral may pose a risk to the patient’s perception of the operative knee, as soft tissue tensioning deviates from the patient’s native anatomy. If an inexperienced surgeon wrongly overstuffed during the surgery, it would accelerate the wear of the prosthesis and the progression of lateral compartment OA [33].

The traditional guidelines of UKA have suggested that patients who have < 10 degrees of varus or < 5 degrees of valgus as suitable candidates for medial or lateral UKA [34]. But with the development of modern UKA techniques, the criteria for UKA candidates have been expanded. Recent evidence even recommended that patients with significant flexion contractures or varus alignment > 15 degrees still could obtain a pre-disease state following UKA and experience excellent outcomes [16, 17]. When we choose the UKA candidates with severe varus deformity, more attention should be paid to other prior limits like age, body mass index, patello-femoral disease, and chondrocalcinosis.

UKA is often compared with HTO [35, 36], especially for patients with severe varus deformity. Patients with varus alignment (HKA ≤ 175°), especially severe extra-articular deformity, may be candidates for HTO, but it is common to see that those patients are often combined with moderate to severe intra-articular OA progression. In this situation, HTO might not be to optimal choice. This study demonstrates that patients with larger angles of alignment change after Oxford UKA are mostly derived from the severe varus group preoperatively, as Group C had the most varus preoperatively, aHKA (*P* < 0.05). And the PROMs of these patients were desirable and not significantly different from patients in other groups (*P* > 0.05).

Paradoxically, modern UKA techniques often implement a limited deformity change to avoid overloading the unaffected compartment, but patients with severe varus deformity are likely to obtain higher angles of perioperative deformity change to restore the original (pre-disease) mechanical axis. However, in this study, we proved that perioperative alignment change was not correlated with the clinical outcome in Oxford UKA patients.

It is a long-standing concept that severe varus deformity is one of the most critical risks to UKA, as it could lead to poorer clinical outcomes postoperatively and eventually increase the prosthesis failure rate [37–39]. Nevertheless, not all of the OA patients with severe varus deformity could meet the inclusion criteria. Only if the patients with predominantly medial compartment osteoarthritis and the severe varus deformity are mostly from the intra-articular side, could these patients with severe varus deformity be beyond the classical indications for UKA as proposed by Scott? [40], be considered for Oxford UKA.

The 2-year survivorship for all group is 100%. This is a cohort study comparing the outcome of UKA in patients with different angles of alignment change. It is a retrospective study, and hence, this was not a randomized controlled trial. This study did not compare against patients with a large angle of varus deformity correction who underwent total knee arthroplasty. However, this is not surprising as many patients would have been considered for total knee replacement instead.

Our study had a few limitations. Use of retrospective data has inherent biases and limitations. In addition, there were differences between groups in terms of radiographic outcomes and demographics, such as age, gender, preoperative coronal alignment, and BMI, which could have a confounding effect on the observed outcomes. A larger sample size and multiple-center study should be conducted, and the patients by different ages, BMI, and activity levels. Moreover, this series of cases was performed at a high-volume center by a single high-volume surgeon, and therefore, the observations of the present study may not be generalizable to lower-volume centers. The other limitation is that the postoperative follow-up and clinical scores assessment at two years is relatively too short, so long-term follow-up data are necessary to verify whether Oxford UKA patients with perioperative great alignment changes would obtain a durable and satisfactory outcome.

Nevertheless, the current study may be the first to evaluate the effect of great varus alignment change on clinical outcome following Oxford UKA arthroplasty. The clinical relevance of this study was to demonstrate that patients with severe varus deformity could tolerate a wide range of perioperative mechanical alignment changes following Oxford UKA arthroplasty to restore a pre-arthritic status. Oxford UKA patients with great perioperative alignment change could obtain a satisfactory clinical outcome. And total knee arthroplasty may not be the only viable option for these patients.

## Conclusions

This study demonstrated that patients could tolerate a wide range of perioperative mechanical alignment changes with favorable patient-reported outcomes at mid-term follow-up. Our study supports Oxford UKA in patients who have the necessary degree of change to achieve a pre-arthritic status. Patients with great varus alignment changes following Oxford UKA arthroplasty did not have rapid progression of lateral compartment osteoarthritis or elevated rates of early UKA loosening. This study has led to a profound and critical thinking of the inclusivity for Oxford UKA arthroplasty, and long-term follow-up is needed to assess the durability of UKA with greater perioperative aHKA changes.

## Data Availability

The datasets used and/or analysed during the current study are available from the corresponding author on reasonable request.
